# Evaluation of Dynamic [^18^F]-FDG-PET Imaging for the Detection of Acute Post-Surgical Bone Infection

**DOI:** 10.1371/journal.pone.0041863

**Published:** 2012-07-30

**Authors:** Tracy L. Y. Brown, Horace J. Spencer, Karen E. Beenken, Terri L. Alpe, Twyla B. Bartel, William Bellamy, J. Michael Gruenwald, Robert A. Skinner, Sandra G. McLaren, Mark S. Smeltzer

**Affiliations:** 1 Department of Radiology, University of Arkansas for Medical Sciences, Little Rock, Arkansas, United States of America; 2 Department of Biostatistics, University of Arkansas for Medical Sciences, Little Rock, Arkansas, United States of America; 3 Department of Microbiology and Immunology, University of Arkansas for Medical Sciences, Little Rock, Arkansas, United States of America; 4 Department of Pathology, University of Arkansas for Medical Sciences, Little Rock, Arkansas, United States of America; 5 Department of Orthopaedic Surgery and Center for Orthopaedic Research, University of Arkansas for Medical Sciences, Little Rock, Arkansas, United States of America; Duke University Medical Center, United States of America

## Abstract

Diagnosing bone infection in its acute early stage is of utmost clinical importance as the failure to do so results in a therapeutically recalcitrant chronic infection that can only be resolved with extensive surgical intervention, the end result often being a structurally unstable defect requiring reconstructive procedures. [^18^F]-FDG-PET has been extensively investigated for this purpose, but the results have been mixed in that, while highly sensitive, its specificity with respect to distinguishing between acute infection and sterile inflammatory processes, including normal recuperative post-surgical healing, is limited. This study investigated the possibility that alternative means of acquiring and analyzing FDG-PET data could be used to overcome this lack of specificity without an unacceptable loss of sensitivity. This was done in the context of an experimental rabbit model of post-surgical osteomyelitis with the objective of distinguishing between acute infection and sterile post-surgical inflammation. Imaging was done 7 and 14 days after surgery with continuous data acquisition for a 90-minute period after administration of tracer. Results were evaluated based on both single and dual time point data analysis. The results suggest that the diagnostic utility of FDG-PET is likely limited to well-defined clinical circumstances. We conclude that, in the complicated clinical context of acute post-surgical or post-traumatic infection, the diagnostic utility accuracy of FDG-PET is severely limited based on its focus on the increased glucose utilization that is generally characteristic of inflammatory processes.

## Introduction

Osteomyelitis is a complicated infection that often requires surgical debridement as well as appropriate, long-term antimicrobial therapy. This is particularly true in chronic infections [1], which are characterized by a compromised blood supply and the formation of necrotic bone [2], [3]. Thus, the definitive detection of infection in its early, acute phase would be ideal. A number of noninvasive imaging modalities have been investigated in this regard [4], [5]. Plain radiographs are capable of detecting infection only after it has progressed to reveal the osteolytic lucency characteristic of chronic infection [6]. Magnetic resonance imaging (MRI) demonstrates higher sensitivity but its ability to detect infection in the setting of post-operative inflammation is limited, particularly in the context of metallic implants. Techniques based on *in vitro* radiolabeling and injection of a patient’s autologous leukocytes are capable of *in vivo* tracking to sites of infection but are hindered by the necessity of blood handling and its associated risks. Three-phase bone scans are exquisitely sensitive for the detection of bone remodeling resulting from multiple etiologies but are limited with respect to distinguishing between infection and other sources of inflammation [4], [6].

Positron emission tomography utilizing 2-deoxy-2-[^18^F]-fluorodeoxyglucose (FDG-PET) has also been investigated as an imaging modality for the diagnosis of osteomyelitis in the post-operative setting [7]–[10]. Some studies have concluded that FDG-PET could be used to differentiate recuperative remodeling from infection as early as 14–21 days post-surgery [8]–[10]. While these results are promising, the transition to a chronic state can occur in as few as 10 days post-infection [3]. This suggests that FDG-PET as it is generally applied in the clinical setting provides definitive diagnostic results only as the infection nears or progresses into the chronic stage. Indeed, based on a clinical study of 21 patients with confirmed osteomyelitis, Kalicke et al. [11] concluded that “in early post-operative follow-up it was impossible to differentiate between post-surgical reactive changes and further infection using FDG-PET”.

Because glucose consumption is increased in metabolically active cells, the uptake of FDG as a glucose analog is increased in rapidly dividing tumor cells and activated inflammatory cells. Thus, the problem with FDG-PET lies in its inability to distinguish between infection and sterile causes of increased glucose uptake. Recent studies have suggested that it might be possible to increase FDG-PET specificity by employing dual-time point imaging [12], [13], but to date this has not been evaluated in a well-defined experimental setting. The goal herein was to use an established rabbit model to comprehensively compare alternative methods for the acquisition and analysis of [^18^F]-FDG-PET data with a specific emphasis on distinguishing between infection and normal recuperative post-surgical inflammation during the therapeutically critical acute phase of infection.

## Materials and Methods

### Rabbit Osteomyelitis Model

All animal procedures were reviewed and approved by the Institutional Animal Care and Use Committee of the University of Arkansas for Medical Sciences. The experiments were carried out in a series of 3 trials, each of which consisted of 12 rabbits (6 infected, 6 uninfected). Thus, a total of 36 male New Zealand white rabbits underwent surgery as previously described [14]. In brief, a 1-cm mid-radial segment was surgically removed, injected with either 10 µl of sterile phosphate-buffered saline (PBS) or an equal volume of PBS containing 10^6^ colony-forming units (cfu) of the *Staphylococcus aureus* osteomyelitis isolate UAMS-1, and replaced in its original orientation prior to wound closure. All animals were imaged as described below before being humanely euthanized 14 days post-surgery, at which time the surgical limb was harvested for bacteriological and histological analysis as previously described [14].

**Figure 1:**
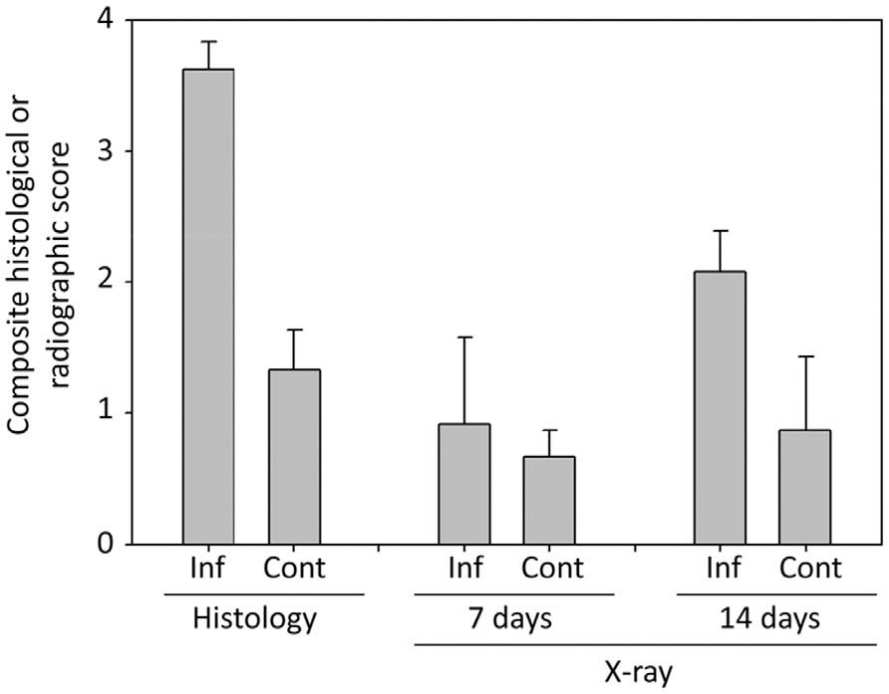
Quantitative description of infection status in each experimental group. Quantitative histological and radiological analyses were done as previously described [14] with the results reported as the average ± the standard deviation for the infected (Inf) and uninfected (Cont) groups. Histological results were obtained only at the completion of the experiment while X-ray studies were performed at both 7 and 14 days.

**Figure 2:**
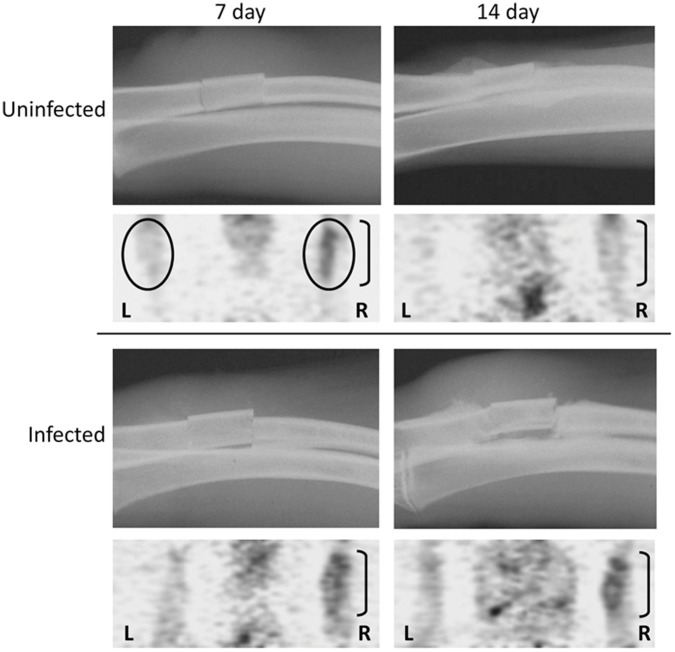
Representative X-ray and FDG-PET images. Representative X-rays of the surgical site are shown for infected and uninfected rabbits as a function of time after surgery. Summed FDG-PET images below each X-ray illustrate uptake in both the surgical (R, surgical site illustrated by bracket) and non-surgical limbs (L) in an infected and an uninfected animal. “Regions of interest” (ROIs) used for measurement of SUV in each limb are illustrated by the circles.

**Figure 3:**
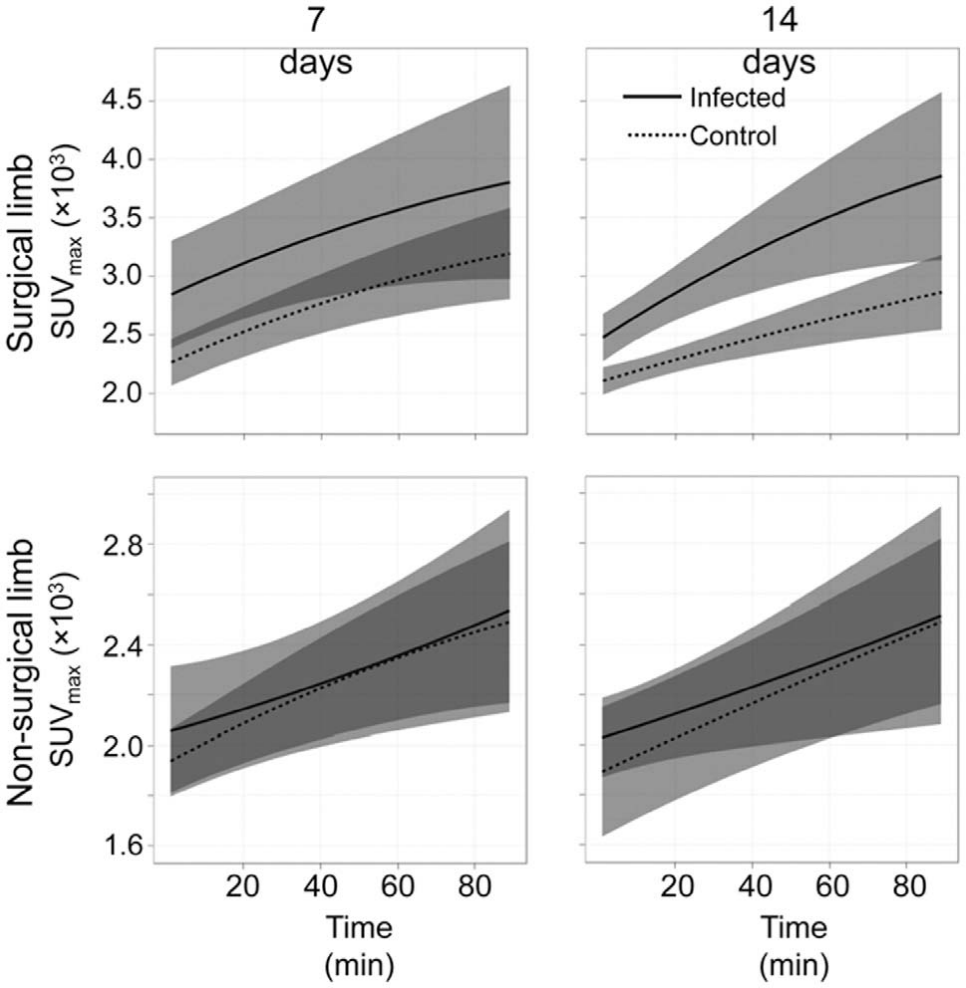
Uptake of FDG over time. Uptake in the surgical (top) vs non-surgical limb (bottom) is shown for the infected and uninfected control groups as a function of time after the administration of FDG. Results for each group obtained 7 and 14 days after surgery are shown as the average SUV_max_ (solid line) ± one standard deviation from the mean (shaded regions). Results represent the averages ± standard deviations obtained after combining all experimental animals in the 1^st^ and 2^nd^ trials (see text).

### Imaging Studies

Radiographs of the surgical limb were taken on post-operative days 7 and 14 using a closed-system AXR small animal radiograph. X-rays were scored by an orthopedic surgeon who was blinded to the infection status of each rabbit and not involved in the analysis of any other data including that obtained by FDG-PET. Scoring was based on evidence of (a) periosteal elevation, (b) sequestration, (c) architectural deformation, and (d) deformation of soft tissue as previously described [14]. Each parameter was scored on a five-point scale (0–4), with 4 representing the most severe evidence of disease.

**Figure 4:**
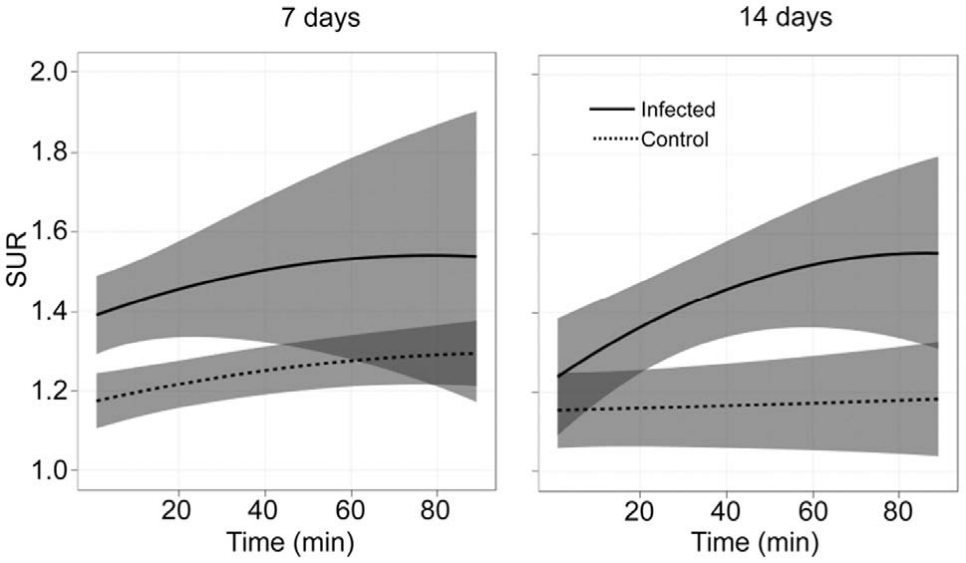
Uptake of FDG over time as a function of SUR. The ratio of SUV_max_ in the surgical vs. non-surgical limbs (SUR) was plotted as a function of time after the administration of FDG. Results for the infected and uninfected control groups 7 and 14 days after surgery are shown as the average SUV_max_ (solid line) ± one standard deviation from the mean (shaded regions). Results represent the averages ± standard deviations obtained from the 3^rd^ experimental trial. Arrows along the axes indicate the timepoint (bottom) and SUR (left) at which the greatest discrimination between the two groups was observed.

Positron emission tomography with [^18^F]-FDG was performed immediately after radiography. Under anesthesia maintained by inhaled isofluorane, approximately 37–74 MBq (1–2 mCi, range 0.9–2.3 mCi) [^18^F]-FDG was injected intravenously into an ear vein. Dynamic acquisition was performed for 90 minutes using a small animal Concorde Focus 220 microPET system equipped with a 14×14 array of lutetium oxyorthosilicate crystals. This allowed for a 19 cm (transaxial) ×7.6 cm (axial) field-of-view and intrinsic spatial resolution of approximately 1.3 mm at the center of the field.

**Table 1:** Diagnostic parameters as a function of FDG-PET evaluation model.

Operating Characteristics	Week 1 Data (N = 12)		Week 2 Data (N = 12)		All Data (N = 24)
	Model 1* c/m# (%)	Model 2 †c/m (%)	Model 1 c/m (%)	Model 2 c/m (%)	Both$ c/m (%)
Sensitivity	5/6 (83)	5/6 (83)	2/6 (33)	4/6 (67)	9/12 (75)
Specificity	6/6 (100)	4/6 (67)	5/6 (83)	6/6 (100)	9/12 (75)
PPV	5/5(100)	5/7 (71)	2/3 (66)	4/4 (100)	9/12 (75)
NPV	6/7 (86)	4/5 (80)	5/9 (56)	6/8 (75)	9/12 (75)
Agreement	11/12 (92)	9/12 (75)	7/12 (58)	10/12 (83)	18/24 (75)

* Model 1: ratio @ 11 mins ≥1.27 classified as infected for week 1 measures.

† Model 2: ratio @ 85 mins ≥1.45 classified as infected for week 2 measures.

$ Both: ratio @ 11 mins ≥1.27 or ratio @ 85 mins ≥1.45 classified as infected.

# *c* is the cell count and *m* is the appropriate marginal total.

FDG-PET data was analyzed by a nuclear medicine physician who was also blinded to the infection status of each rabbit and unaware of radiographic scoring results. Raw PET data was reconstructed using filtered backprojection and binned into 2-minute increments beginning at time 0. Transverse, coronal, and sagittal reconstructed images were interpreted using AMIDE medical imaging software. Volumes-of-interest encompassing the surgical site in the right radius were drawn on reconstructed transverse images, and maximum standardized uptake values corrected for rabbit weight (in kg, range 2.6–3.4 kg) (SUV_bw,max_; hereafter referred to as SUV) were calculated at each timepoint. The ratio (SUR) of SUV in the surgical limb to SUV in the non-surgical limb was also calculated for each animal at each data acquisition timepoint, *t*, based on the formula SUR*t*  =  [(SUV right, *t* - SUVleft, *t*)/SUV left, *t*]. Additionally, the change in SUV at the surgical site (ΔSUV*t*1:*t*2) was calculated based on the formula ΔSUV*t*1:*t*2 =  (SUV right, *t*2– SUV right, *t*1)/SUV *t*1 where *t1* and *t2* are early and late timepoints, respectively, within the 90-minute data acquisition period. For simplicity in presenting results, we define SUR*t*  =  ΔSUV0:*t*.

**Figure 5:**
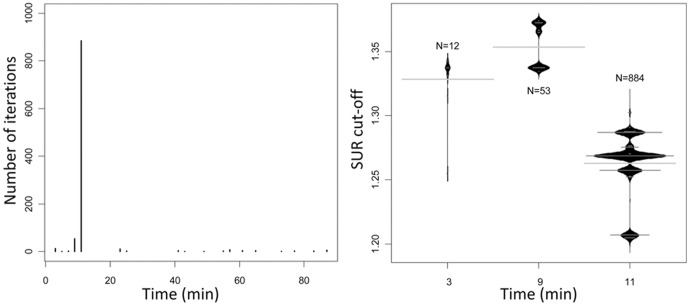
Confirmation of optimal 7 day diagnostic parameters. Left: Results illustrate the number of 1000 randomly-generated datasets in which the indicated timepoint after the administration of FDG exhibited maximum discrimination between the infected and uninfected experimental groups. Right: Results illustrate the proportion of “cut-off” ratios (surgical vs. non-surgical limb) that exhibited optimal discrimination at the alternative timepoints shown. Note that >850 of 1000 “iterations” found the optimum timepoint to be 11 minutes after administration of FDG (left) and that the optimal SUR cutoff in the majority of these was ∼1.27.

**Figure 6:**
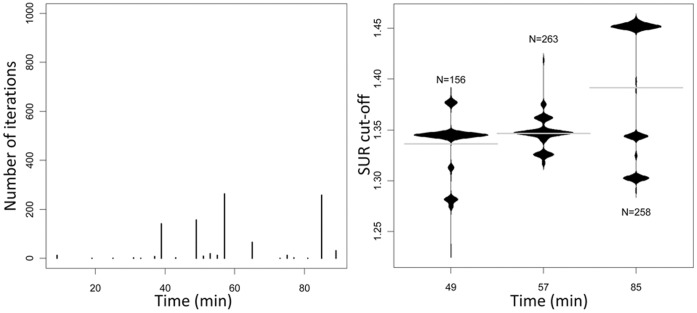
Identification of optimal 14 day diagnostic parameters. Left: Results illustrate the number of 1000 randomly-generated datasets in which the indicated timepoint after the administration of FDG exhibited maximum discrimination between the infected and uninfected experimental groups. Right: Results illustrate the proportion of “cut-off” ratios (surgical vs. non-surgical limb) that exhibited optimal discrimination at the alternative timepoints shown. Note that the number of “iterations” in which the greatest discrimination was observed were comparable when the assay was done 57 or 85 minutes after the administration of FDG and that the optimal SUR cutoff differed between these two timepoints.

**Figure 7:**
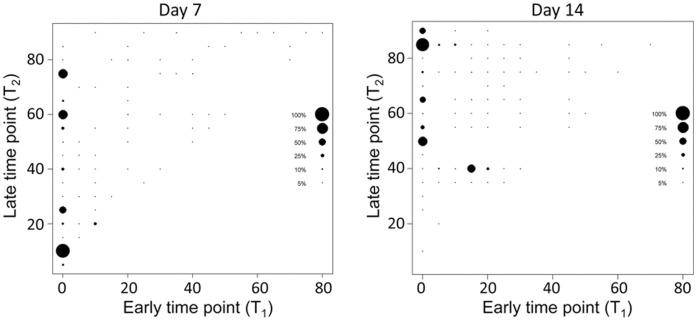
Best classifiers of infection status at week 1 and week 2 for all data. Classifiers of infection status over 1000 iterations are plotted, with frequency of classifier identification as the best in any given iteration indicated by size of the dot for that classifier. On day 7, the 5 best classifiers all occurred when *t_1_ = 0*; that is, at a single timepoint SUR, with the single best classifier being <15 min after the administration of FDG. On day 14, all but one (*δ*SUV at *t_1_* = 15 min and *t_2_* = 40 min) of the 5 best classifiers of infection status were a single timepoint SUR, with the single best classifier being >85 min after the administration of FDG.

### Statistical Analyses

Data from the first two trials was examined individually to evaluate the variance within and between experimental trials. Because the variance between trials was small by comparison to the variance within each trial, data from these experiments was combined for further analysis. Mixed effect models were used to examine SUV and SUR trends over scan time. Separate analyses were performed for data obtained at day 7 and day 14. The fixed effects terms of the models included a factor representing infection status, linear and quadratic scan time terms, and terms representing the interaction between infection status and scan time, both linear and quadratic. Means and 95% confidence intervals for each experimental group were calculated for each scan time. An α-level of 0.05 was used to determine the statistical significance of the interaction and infection status effects.

Classification and regression tree (CART) methodology was used to generate simple prediction models based on SUR values. The SUR measures were recorded every 2 minutes for 90 minutes, producing 45 measurements per rabbit per week of the experiment. Data from trials 1 and 2 were used to create prediction models used to evaluate data obtained from rabbits during trial 3.

To assess how generalizable the simple models were, data from all three trials was combined and 12 rabbits randomly selected and held back as a “test” set. Data from the remaining rabbits were used as a “training” set on which the CART methodology was applied to define a simple model (i.e. the best scan time and SUR ‘cut point’ for distinguishing between infected and uninfected rabbits). This was repeated 1000 times to determine which scan times were most often selected as well as the associated SUR value identified as the cut-point.

The CART analysis described above was also expanded to include ΔSUR*t1:t2* measures as potential predictors to determine whether analysis based on multiple timepoints offered a diagnostic advantage. The process was again repeated 1000 times and, after each iteration, the ΔSUR*t1:t2* resulting in the “best” split was captured.

## Results

Due to anesthesia complications during scanning, data was obtained for the entire 90 min scan period at day 7 for only 21 of 24 rabbits and at day 14 for only 20 of 24 rabbits included in trials 1 and 2. These failures included both infected and uninfected rabbits, with a complete dataset available for at least 10 rabbits from each group on each scan day.

Bacteriological and histological analysis confirmed the infection status of all rabbits based on the isolation of the specific strain used to initiate the infection and the presence of Gram-positive intra-osseous cocci respectively (data not shown). Additionally, overall histopathology scores were significantly higher in the infected vs. the uninfected experimental groups (Fig. 1). Radiographs revealed little difference between the infected and control groups 7 days after surgery, but the two groups diverged thereafter, with the overall difference reaching statistical significance (p<0.001) by day 14 (Fig. 1).

Representative summed FDG-PET images obtained 7 and 14 days after surgery are illustrated in Fig. 2 in the context of the comparative X-rays. FDG uptake at the surgical site increased over the entire 90-min imaging period in both the infected and uninfected experimental groups, but the average SUV at the surgical site in the infected rabbits was consistently higher than the average SUV in the uninfected controls throughout this imaging period at both 7 and 14 days (Fig. 3). In contrast, the average SUV was comparable in both experimental groups in the non-operative limb. During the early imaging period (post-injection time <20 min), SUV was higher at the operative site in infected rabbits at 7 days than at 14 days, but was essentially equivalent after 90 minutes.

Differences between the infected and uninfected groups, and differences within the infected group on day 7 vs. day 14, were also apparent when the analysis was done based on SUR rather than absolute SUV (Fig. 4). In general, the overall patterns confirmed that uptake was delayed at day 14 by comparison to day 7. The greatest discrimination between the two groups on day 7 occurred 11 min after administration of FDG, while on day 14 the greatest discrimination was observed at 85 min (Fig. 4). The SUR “cut point” that offered the greatest discrimination between the infected and uninfected groups also differed, with 1.27 being optimal at day 7 and 1.45 being optimal at day 14.

Based on these results, we carried out a 3^rd^ independent experiment in which the infection status of the animal was predicted using two models. Model 1 defined an infected animal as one having an SUR ≥1.27 based on data obtained 11 min after administration of FDG and Model 2 defined an infected animal as one having an SUR ≥1.45 based on data obtained 85 min after administration of FDG. All diagnostic parameters were considerably higher when Model 1 was applied to data obtained 7 days after surgery and, conversely, when Model 2 was applied to data obtained 14 days after surgery. When Model 1 was used to analyze data obtained 14 days after surgery, or when Model 2 was used to analyze data obtained 7 days after surgery, all diagnostic parameters were considerably lower (Table 1).

CART analysis done with data from day 7 rabbits included in all 3 trials showed that Model 1 parameters (cut-point ≥1.27 at 11 min) offered the greatest discrimination in >88% of 1000 random iterations (Fig. 5). The results obtained when Model 2 was applied to data derived at day 14 were more ambiguous in that a significant proportion of the 1000 iterations suggested alternative cut-points derived at alternative times after the administration of [^18^F]-FDG (Fig. 6). However, all of these alternative parameters were chosen less frequently than those employed in Model 2, and this suggests that the alternative time points for analysis are unlikely to result in an improved diagnostic outcome.

To determine whether dual-time point analysis would allow for better discrimination between infected and uninfected animals, one-step CART analysis was extended to include comparisons with continuous values of *Δ*SUV. To simplify the analysis, data was examined at 5 min intervals throughout the 90-minute scan period and was pooled, and the 5 parameters yielding the best classification of infection status were identified for day 7 and day 14. On day 7 post-surgery, the best classifiers were all single time point SURs (*t1* = 0) (Fig. 7) rather than any ΔSUV. On day 14, 4 of the 5 best classifiers were single timepoint SURs (*t1* = 0) (Fig. 7). The single timepoint most often found to offer to the greatest discrimination differed on day 7 vs. day 14, with 10 min being the single best option for the former and 85 min being the single best option for the latter (Fig. 7).

We next analyzed the data from the 3^rd^ experiment using an ‘either/or’ approach. Specifically, data obtained from all 12 rabbits on both scan days was combined into a single dataset (*n* = 24). This dataset was then analyzed based on defining an infected rabbit as one in which either the Model 1 or Model 2 parameters were met and, conversely, defining an uninfected rabbit as one in which neither of these parameters were met. Using this approach, all diagnostic parameters were found to be 75% (Table 1).

## Discussion

All forms of bone infection present a therapeutic challenge, but this challenge is greatly magnified once the infection has progressed from an acute to a chronic state. Therefore, it is important to develop non-invasive diagnostic techniques capable of detecting infection in its earliest stages. The most definitive diagnostic method is microbiological culture, but in the specific context of bone and implant-associated infection this requires access to the site, and this can only be achieved using invasive procedures. Because such procedures themselves offer an opportunity for infection, it is imperative that they be employed judiciously. This accounts for the need to develop non-invasive diagnostic imaging modalities capable of both detecting infection in its early stages when it is more therapeutically amenable and distinguishing between infection and sterile forms of inflammation. In a previous study using a rabbit model to examine the diagnostic utility of static FDG-PET in the clinical context of post-surgical infection, we found that FDG-PET did offer promising diagnostic utility at least by comparison to plain-film X-ray, but only after the infection had progressed to a chronic stage as defined in the specific context of this rabbit model [9], [14]; prior to that time, it was not possible to reliably distinguish between infection and recuperative post-surgical inflammation.

In this report we attempted to build on this previous work to determine whether the diagnostic utility of FDG-PET during the critical acute phase of infection could be increased by employing alternative methods of data acquisition and analysis including dynamic imaging based on changes in SUV over time. We found that FDG-PET could distinguish between infection and normal post-operative healing with >90% accuracy as early as 7 days after surgery, which is potentially significant in that this is within the time frame of acute infection when it may still be possible to resolve the infection without additional surgical intervention [2], [3]. However, achieving this was dependent on the specific analysis method employed, with the critical issue being assessment of uptake within 10–12 min after FDG administration.

This is in contrast to the optimum method defined by our experiments done 14 days after surgery, which demonstrated that the best discrimination is obtained using data acquired ∼85 min after the administration of FDG. In this context it is important to note that X-rays also provided a reliable indication of infection status on day 14. To the extent that our previous studies using this same model demonstrated that X-rays provided reliable diagnostic information only after the infection had progressed to a chronic stage [9], [14], this suggests that the infected rabbits included in this study had developed changes characteristic of chronic infection by day 14. This relatively rapid time frame presumably reflects our use of the high infectious dose, an approach we employed to ensure that the infection was established in all experimental animals. Thus, when taken together, the observation that the optimum imaging time was significantly delayed by comparison to studies done 7 days after infection reflects delayed uptake owing to secondary changes related to chronic infection.

These results demonstrate that the diagnostic utility of FDG-PET is highly dependent on the status of the underlying infection and the specific method by which it is applied. This could be useful information in certain clinical settings, most notably in patients with findings concerning for infection in the early post-operative or post-traumatic period where the infection is likely to be in its acute phase and in which the timing of inoculation is known. This also raises the possibility that FDG-PET could be used not only to detect infection but also to stage the infection as an important consideration in planning the therapeutic approach. However, when we applied our optimized methods independently of the post-surgical interval, the diagnostic accuracy of FDG-PET was significantly reduced, thus confirming that FDG-PET has limited diagnostic utility when the length of time since infection onset is unknown. Additionally, while dynamic FDG-PET is now clinically feasible owing to improvements in instrumentation, our data does not support the hypothesis that dual-time point imaging offers a significant advantage over single time point imaging in osteomyelitis of unknown duration.

Finally, while certain of these results appear promising under well-defined clinical conditions, it must be noted that these results are based on a well-controlled experimental system with age-matched animals maintained on the same diet, and it is certain that this represents a best-case scenario by comparison to the diversity that would be encountered in individual patients. Indeed, given this diversity, it would be difficult to envision that infections would progress in a consistent timeframe in all patients. Thus, we conclude that the diagnostic utility of FDG-PET is likely to be limited beyond very well-defined clinical circumstances irrespective of the manner in which it is applied. This suggests that imaging tracers specific for bacteria, rather than host physiological features like inflammation, are likely to be of greater diagnostic value.
